# Let-7, Mir-98 and Mir-181 as Biomarkers for Cancer and Schizophrenia

**DOI:** 10.1371/journal.pone.0123522

**Published:** 2015-04-09

**Authors:** Emmanouil Rizos, Nikolaos Siafakas, Eleni Katsantoni, Eleni Skourti, Vassilios Salpeas, Ioannis Rizos, James N. Tsoporis, Anastasia Kastania, Anastasia Filippopoulou, Nikolaos Xiros, Demetrios Margaritis, Thomas G. Parker, Charalabos Papageorgiou, Vassilios Zoumpourlis

**Affiliations:** 1 National and Kapodistrian University of Athens, Medical School, 2nd Department of Psychiatry, University “ATTIKON” General Hospital, Athens, Greece; 2 National and Kapodistrian University of Athens, Medical School, Microbiology Laboratory, University “ATTIKON” General Hospital, Athens, Greece; 3 Biomedical Research Foundation, Academy of Athens, Hematology-Oncology Division, Athens, Greece; 4 Unit of Biomedical Applications, Institute of Biology, Medicinal Chemistry & Biotechnology, National Hellenic Research Foundation, Athens, Greece; 5 National & Kapodistrian University of Athens, 2nd Cardiology Department, University General Hospital “ATTIKON”, Athens, Greece; 6 Keenan Research Centre. Li Ka Shing Knowledge Institute for Biomedical Science, St. Michael’s Hospital, Toronto, Canada; 7 Department of Informatics, Athens University of Economics and Business, Athens, Greece; 8 Second Department of Propaedeutic Internal Medicine, Oncology Unit, Attikon University Hospital, Athens, Greece; 9 Medical School, Democritus University of Thrace, University General Hospital of Alexandroupolis, Department of Psychiatry, Alexandroupolis, Greece; The Nathan Kline Institute, UNITED STATES

## Abstract

Recent evidence supports a role of microRNAs in cancer and psychiatric disorders such as schizophrenia and bipolar disorder, through their regulatory role on the expression of multiple genes. The rather rare co-morbidity of cancer and schizophrenia is an old hypothesis which needs further research on microRNAs as molecules that might exert their oncosuppressive or oncogenic activity in the context of their role in psychiatric disorders. The expression pattern of a variety of different microRNAs was investigated in patients (N = 6) suffering from schizophrenia termed control, patients with a solid tumor (N = 10) and patients with both schizophrenia and tumor (N = 8). miRNA profiling was performed on whole blood samples using the miRCURY LNA microRNA Array technology (6th & 7th generation). A subset of 3 microRNAs showed a statistically significant differential expression between the control and the study groups. Specifically, significant down-regulation of the let-7p-5p, miR-98-5p and of miR-183-5p in the study groups (tumor alone and tumorand schizophrenia) was observed (p<0.05). The results of the present study showed that let-7, miR-98 and miR-183 may play an important oncosuppressive role through their regulatory impact in gene expression irrespective of the presence of schizophrenia, although a larger sample size is required to validate these results. Nevertheless, further studies are warranted in order to highlight a possible role of these and other micro-RNAs in the molecular pathways of schizophrenia.

## Introduction

Schizophrenia and cancer share a broad spectrum of clinical phenotypes and a complex biological background, implicating a large number of genetic and epigenetic factors [1]. Moreover, these two conditions seem to share a suspected aetiology that includes the influence of several transcriptomic components, suggesting that epigenetic regulation of gene expression through the action of miRNAs may represent a central common feature in the low co-incidence of schizophrenia and cancer [2]. There is an old hypothesis about the rather rare co-incidence of schizophrenia and cancer. Previous, population-based studies have shown the decreased incidence of carcinogenesis in schizophrenic patiens [3]. Since then, several speculations have been made regarding the possible molecular basis for these observations, but no specific results have been recorded so far.

There has been an increasing interest on the role of microRNAs as regulatory factors in the aetiology and pathophysiology of major psychiatric disorders, such as schizophrenia, autism, bipolar disorder etc [4–6]. MicroRNAs are a class of small, non-coding RNAs that play an important role in various biological processes [7], including both psychiatric disorders and carcinogenesis. miRNAs seem to have a post-transcriptional control of gene expression, resulting in gene silencing or gene expression stimulation [8]. Recent studies have revealed specific roles of miRNAs in brain development [9,10], in neurodevelopmental disorders [11] and in long-term memory storage [12]. The role of miRNAs in carcinogenesis has also been established in a large number of reports. It has recently been revealed that 98 out of 186 miRNA genes located in cancer-associated genomic regions may frequently be found in different types of tumors, whereas altered expression of let-7 and miR-155 in lung cancer was correlated with the survival of patients [13,14].

There is no satisfactory evidence so far regarding the possible regulatory role of miRNAs in the co-incidence and co-morbidity of schizophrenia and cancer. In our previous study, a significant down-regulation of miR-183 in a group of patients with both schizophrenia and cancer was observed in comparison with schizophrenic patients, providing a first indication that miR-183 may play a role in regulating the expression of other genes with onco-suppressor activity [15]. In the present study, a larger sample size that now included another study group of patients with cancer only was investigated regarding expression of many different RNAs, in an attempt to provide further insight about the possible molecular explanation of the low incidence of cancer risk among schizophrenic patients.

## Materials and Methods

### Subjects

**Fig 1:**
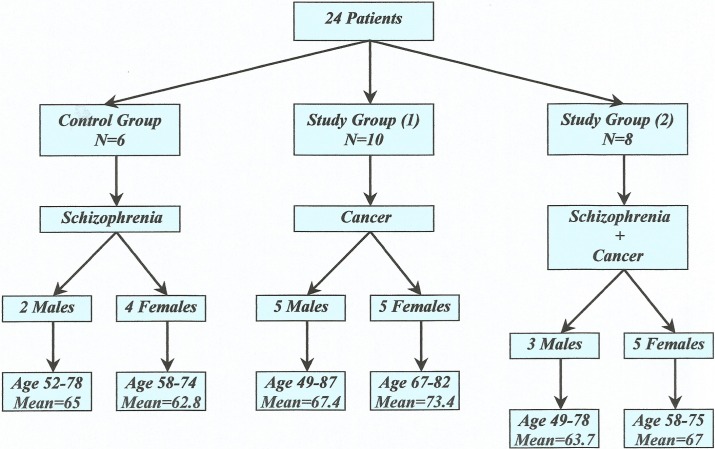
Control and Study groups of the study. No statistically significant difference was found between the mean ages of the control and the study groups (student's t-test, *p* = 0.36).

Twenty-four patients were recruited from the Psychiatric Hospital of Attica and from the Oncology Outpatient Clinic of the “ATTIKON” University General Hospital from February 2011 till December of 2012. A schematic presentation of both control and study groups of patients is shown in **Fig 1**, whereas more detailed clinical information is provided in **Table 1**. These patients were assessed by SCID-IV [16] and by Positive and Negative Syndrome subscales (PANSS) [17]. Exclusion criteria included a history of any neurological disease and current misuse or dependence in the preceding 6 months as defined by DSM-IV [18]. All patients were in a stabilized psychological state and the medication was kept unchanged during the last 6 months at least. The diagnosis of cancer was made according to the medical records of the patient.

**Table 1:** Clinical information about all patients with schizophrenia and/or cancer that were included in the present study.

***Control Group***				***Study Group (1)***						
***Patient***	***Sex***	***Age***	***Age of Schizophrenia Diagnosis***	***Patient***	***Sex***	***Age***	***Age of Cancer Diagnosis***	**Site of Cancer**	***Stage at Diagnosis***	***Stage of Cancer During Sampling***
No. 1	M	52	39	No. 1	F	67	63	Uterus Carcinoma	I	IV
No. 2	F	58	40	No. 2	F	76	74	Urothelial Cell Carcinoma	II	IV
No. 3	F	60	34	No. 3	F	67	61	Ovary Carcinoma	IIIA	IV
No. 4	F	58	38	No. 4	M	49	48	Prostate Adenocarcinoma	IIA	IV
No. 5	F	74	45	No. 5	M	65	60	Prostate Adenocarcinoma	IIA	IV
No. 6	M	78	41	No. 6	M	68	62	Prostate Adenocarcinoma	IIB	IV
				No. 7	M	87	84	Squamous Cell Lung Carcinoma	II	III
				No. 8	F	70	68	Endometrial Sarcoma	II	IV
				No. 9	F	87	82	Breast Adenoacrcinoma	II	IV
				No. 10	M	68	66	Non—Small Cell Lung Carcinoma	II	IV
***Study Group (2)***										
***Patient***	***Sex***	***Age***	***Age of Schizophrenia Diagnosis***		***Age of Cancer Diagnosis***		***Site of Cancer***		***Stage at Diagnosis***	***Stage of Cancer During Sampling***
No. 1	F	75	43		72		Colorectal Cancer		IIIB	IV
No. 2	F	72	45		69		Colorectal Cancer		IIIA	IV
No. 3	M	78	39		71		Prostate Carcinoma		II	III
No. 4	F	66	34		52		Breast / Thyroid Cancer		IIA/I	IV/I
No. 5	M	64	37		63		Urothelial Cell Carcinoma		III	IV
No. 6	F	58	39		56		Laryngeal Carcinoma		II	III
No. 7	M	49	47		42		Thyroid Cancer		I	I
No. 8	F	64	41		63		Lung Carcinoma		III	IV

The control group consisted of 6 patients (M/F: 2/4, age 48–78 years) suffering from chronic schizophrenic disorder, which had no evidence of cancer disease (**Fig 1**). Inclusion criteria for the control group were body weights within 10% of an appropriate body mass index, no other serious diseases except schizophrenia, no clinically significant abnormal laboratory values and no pathological findings on thorough clinical examination. Additionally, all individuals in the control group had no history of hypersensitivity (asthma, urticaria and eczema), autoimmune disorders such as systemic lupus erythematosus, uncontrolled hypertension or serious heart, lung, liver or renal condition.

The study group consisted of 18 patients (M/F: 8/10, age 49–87 years) suffering either from both schizophrenia and cancer (N = 8), or cancer only (N = 10) (**Fig 1**). Exclusion criteria included a history of any neurological disease and current misuse or dependence in the preceding 6 months, as defined by DSM-IV [18]. All patients were in a stabilized psychological state and the medication was kept unchanged during the last 6 months at least. Diagnosis of cancer was made according to the medical records of the patient. The patients suffering from cancer only had no history of psychiatric symptoms and they had a negative familial history of any major psychiatric disorder.

All schizophrenic patients were in a stabilized psychological state and their medication was kept unchanged during the last 6 months at least. Smoking history but not recent history (>1 year) of alcoholism, recreational drugs or drug addiction were allowed in the inclusion criteria for both groups. All female participants were tested and found not to be pregnant. All schizophrenic patients were on regular medical treatment with antipsychotics. All participants were able to communicate effectively, were informed about the nature of the study and provided written informed consent. The study was approved by the institutional review board and ethics committee of both participating hospitals (University General Hospital “ATTIKON”, Psychiatric Hospital of Attica) and was conducted in accordance with Good Clinical Practice principals and applicable local regulations. The schizophrenic patients were clinically followed up for one year after blood collection for the present study and showed no evidence of cancerous disease.

### Sample collection and preservation

miRNA profiling for each patient was performed on whole blood samples. Preservation of the gene expression status of the samples was achieved by collecting 500μl of whole blood from each patient to an RNAprotect Animal Blood Tube (Qiagen, Hilden, Germany). Following gentle inversion, the tubes were incubated for 2h at ambient temperature, according to the manufacturer's instructions, in order to allow for efficient cell lysis. All tubes were then stored at -80°C prior to RNA purification.

### RNA purification, quality control and miRNA array profiling

Total RNA purification that contained miRNA was carried out using the RNeasy Protect Animal Blood kit (Qiagen). All experiments that included sample RNA quality control, miRNA profiling and analysis were conducted by Exiqon Services (Vedbaek, Denmark). Briefly, RNA quality control and measurement were carried out using an Agilent 2100 Bioanalyzer and a nanodrop instrument. 300ng of total RNA from both sample and reference were then labeled with Hy3 and Hy5 fluorescent label, respectively, using the miRCURY LNA microRNA Hi-Power Labeling Kit, Hy3/Hy5 (Exiqon, Denmark), following the procedure described by the manufacturer. The Hy3-labeled samples and a Hy5-labeled reference RNA sample were mixed pair-wise and hybridized to the miRCURY LNA microRNA Array 7th (Exiqon, Denmark), which contains capture probes targeting all microRNAs for human, mouse or rat registered in the miRBASE 18.0. The hybridization was performed according to the miRCURY LNA microRNA Array Instruction manual using a Tecan HS4800 hybridization station (Tecan, Austria). Following hybridization the microarray slides were scanned and stored in an ozone free environment (ozone level below 2.0 ppb) in order to prevent potential bleaching of the fluorescent dyes. The miRCURY LNA microRNA Array slides were scanned using the Agilent G2565BA Microarray Scanner System (Agilent Technologies, Inc., USA) and the image analysis was carried out using the ImaGene 9 (miRCURY LNA microRNA Array Analysis Software, Exiqon, Denmark). The quantified signals were background corrected (Normexp with offset value 10) [19] and normalized using the global Lowess (LOcally WEighted Scatterplot Smoothing) regression algorithm. Spike-in controls were added in various concentrations in both the Hy3 and the Hy5 labeling reactions to evaluate the labeling reaction, hybridization, and the performance of the array experiment.

### Data analysis (unsupervised and expression analysis)

Principal Component Analysis (PCA) was applied in order to explore the naturally arising sample classes based on the expression profile. A heat map diagram was produced that showed the results of a two-way hierarchical clustering of microRNAs and samples. The clustering was done using the complete-linkage method together with the euclidean distance measure.

The statistical significance of the relative expression of miRNAs between the control and the study groups was determined using the Student's t-test. Furthermore, the Benjamini-Hochberg multiple testing adjustment method was applied to the *p*-values for the control of possible false positive results.

The results of the microarray analysis were submitted to Gene Expression Omnibus and assigned the accession number GSE65143.

## Results

**Fig 2:**
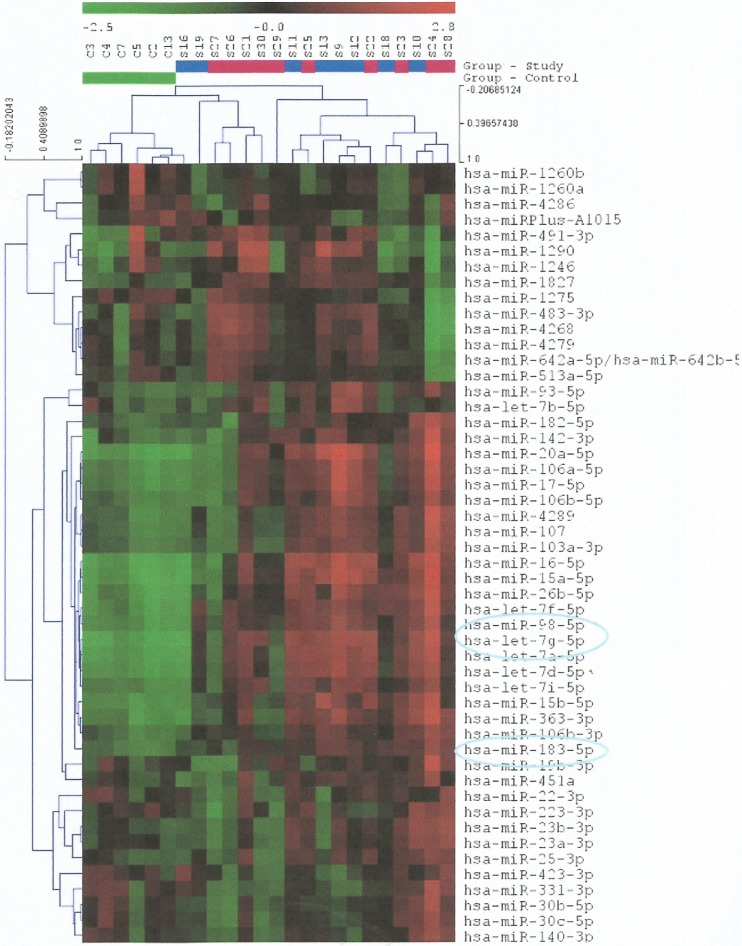
Heat map diagram showing the expression of the 50 miRNAs with the highest standard deviation in all samples. The color scale illustrates the relative expression level of miRNAs and specifically, red color represents an expression level below the reference channel, whereas green color represents an expression higher than the reference. Each row represents a microRNA and each column represents a sample. The microRNA clustering tree is shown on the left. The control and study groups are clearly indicated with different colours. Samples S9-S13, S16, S18 & S19 correspond to the study group of patients with schizophrenia and tumor formation, whereas samples S21-S30 correspond to the study group of patients with tumor formation only. The 3 miRNAs, the expression of which was found to be significantly higher in the samples of the control group of patients, are also indicated.

**Fig 3:**
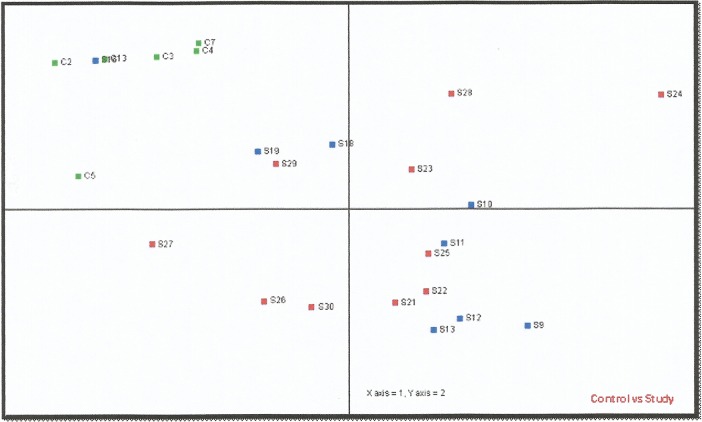
PCA plot of the different sample classes. The principal component analysis was performed on all samples, and on the top 50 microRNAs with the highest standard deviation. The normalized log ratio values have been used for the analysis. The features have been shifted to be zero centered, (i.e. the mean value across samples is shifted to 0) and scaled to have unit variance (i.e. the variance across samples is scaled to 1) before the analysis. Samples C2-C5, C7 & C13 correspond to the control group of patients, whereas sample codes beginning with S correspond to the 2 subsets of the study group, designated and colored separately as in Fig 2.

In total, 322 different miRNAs were analyzed by the miRCURY LNA miRNA Array. **Fig 2**shows a heat map diagram that depicts the expression of the 50 miRNAs with the highest standard deviation on all samples. Statistical analysis of the results showed, first of all, that there were no significant differences in the expression profiles between the 2 study groups (patients with schizophrenia and tumour formation and patients without schizophrenia but with tumour formation), as also depsicted in the PCA plot of **Fig 3.** However, the analysis identified let-7p-5p, miR-98-5p and miR-183-5p with a highly significant differentiation in their expression profile (*p*<0.05, following the application of the Benjamini-Hochberg multiple testing adjustment method) between the control group (patients with schizophrenia but without tumour formation) and both study groups (patients with/without schizophrenia, but with tumour formation). These 3 differentially expressed microRNAs were down-regulated in both study groups, as shown in **Fig 2**.

## Discussion

It is generally known that cancer cells show a decrease in apoptosis levels, whereas neurodegenerative diseases, such as schizophrenia, are marked by an increase in apoptosis. During the present study there was an attempt to determine whether the possibly poor association between cancer and schizophrenia may be associated with certain miRNA expression profiles. These data provided preliminary insight about the differential expression of 3 miRNAs in correlation with cancer only and not with both schizophrenia and cancer, contrary to what was initially expected, although a greater sample size of patients with schizophrenia and/or cancer is still needed in order to obtain more representative conclusions. Specifically, a statistically significant down-regulation of the let-7g-5p, miR-98-5p and miR-183-5p in the study group of patients (patients with solid tumors and patients with schizophrenia and tumor) was observed (p<0.05), providing an indication about their role in the incidence of cancer. High expression levels of these miRNAs may lead to high levels of apoptosis and, thus, enable the incidence of schizophrenia. However, the observed low levels of expression of the same miRNAs in a certain group of patients with schizophrenia and cancer, possibly causing a decrease in apoptosis levels and thus, making them prone to the development of malignant tumors, may indicate development of schizophrenia due to alterations in molecular pathways independent of these miRNAs. Additionally, the neurodevelopmental and neurotoxicity theory has a dominant role on their pathophysiology and thus, down-regulation of let-7, miR-98 and miR-183 may provide a possible molecular explanation.

Let-7 is a miRNA that has never been studied in relation to neurological disorders, such as schizophrenia. However, regarding Parkinson’s Disease (PD), it is mentioned that in a *Caenorhabditis elegans* model let-7 is down-regulated in strains bearing a typical mutation in alpha-synuclein gene (A53T) and in strains with mutations in the pdr-1 gene [20]. Moreover, increased levels of let-7 were found to attenuate pathogenic LRRK2 effects, a molecule which is very frequently mutated in both familial and sporadic PD [21]. In Alzheimer’s disease (AD), APP-like-1 expression was shown to be modulated by the activity of let-7 family [22]. In another study, let-7 was found to activate Toll-like receptor 7 and subsequently cause neurodegeneration [23]. Regarding the implication of let-7 in cancer, it has been mentioned that this miRNA inhibits cell invasion and migration in non small cell lung cancer (NSCLC) [24]. Moreover, a recent study highlights the implication of let-7 in sensitized fluorouracil-resistant human hepatoma cells [25]. Furthermore, low expression of let-7 predicts poor prognosis in patients with multiple cancers [26]. According to another study, let-7 downregulates STAT3 phosphorylation in pancreatic cancer cells by increasing SOCS3 expression [27].

miR-183 is a miRNA that has never been correlated with neurodegenerative diseases in the past, with the exception of a recent study where the possible suppressive role of miR-183 in the neurobiological pathways involved in schizophrenia was highlighted [15]. Mir183 has recently been implicated in modulation of different apoptotic and autophagic stages through regulation of apoptosis and autophagy-related genes [28–30]. In specific, knock-down of mir-183 expression can induce autophagic cell death in medullary thyroid cancer, through regulation of some tumor suppressive signaling pathways, indicating that mir-183 may be an attractive therapeutic target [31]. The oncogenic role of miR183 has also been revealed in a recent study by targeting the transcription factor EGR1 and promoting tumor cell migration in different types of cancer, such as sarcomas and colon tumors [32]. Therefore, it seems that mir-183 in the present study might have played a rather tumor suppressor role, by probably activating the expression of tumor suppressor genes that control cell differentiation or apoptosis, as previously described [33].

The expression profile, as well as the functional role of miR-98 in neurological diseases, such as schizophrenia, has not been elucidated yet. The only reference to such an association has been made in a recent study, where expression of miR-98 was up-regulated in cases of prenatal stress altering, thus, the inflammatory responses in the brain [1]. In AD, miR-98 displayed significantly different expression levels in patients compared with controls [34]. miR-98 has also been shown to target molecules that have been associated with different forms of cancer, such as HMGA2, which exerts a transforming effect on lung cancer cells [35], IRS2, a protein that is up-regulated in colorectal cancer that leads to an increase in wnt signaling [36] and IGF2BP which has been documented to promote hepatocellular carcinoma [37]. Other molecules that are involved in tumorigenesis and are targeted by miR-98 include N-Ras [38], SNAP23 [39], IMPDH1 [40] and many others.

As previously stated, the rather small sample size possibly constitutes a limiting factor of the present study. However, it must be stated that, in the light of the severe difficulty to recruit patients exhibiting schizophrenia and cancer as a comorbidity, this was the first clinical study that attempted to provide some preliminary insight about the possible low risk of cancer in schizophrenic patients by analysing a large number of miRNAs in patients with both diseases and patients with either schizophrenia or cancer only. Moreover, the level of expression of miRNAs was analyzed in blood and not in the brain, representing, thus, an indirect analysis of brain miRNAs expression levels and as a consequence, data about a general genetic predisposition about the implication of let-7, miR-98 and miR-183 in the co-morbidity of schizophrenia and cancer were collected.

In conclusion, the present study further reinforced the important role of let-7, miR-98 and miR-183 in carcinogenesis, but no miRNAs were found to be involved in the co-incidence of cancer and schizophrenia, as was the objective. Further studies with larger sample sizes are still warranted in order to investigate the role of miRNAs and other molecular pathways in the rather low co-incidence of cancer and schizophrenia.

## References

[1] Zucchi FC, Yao Y, Ward ID, Ilnytskyy Y, Olson DM, Benzies K, et al Maternal stress induces epigenetic signatures of psychiatric and neurological diseases in the offspring. PLoS One. 2013;8: e56967 10.1371/journal.pone.0056967 23451123 PMC3579944

[2] Petronis A. Epigenetics as a unifying principle in the aetiology of complex traits and diseases. Nature. 2010;465: 721–727. 10.1038/nature09230 20535201

[3] Chou FHC, Tsai KY, Su CY, Lee CC. The incidience and relative risk factors for developing cancer among patients with schizphrenia: a nine-year follow-up study. Schizophrenia Res. 2011;129: 97–103. 10.1016/j.schres.2011.02.018 21458957

[4] De Smaele E, Ferretti E, Gulino A. MicroRNAs as biomarkers for CNS cancer and other disorders. Brain Res. 2010;1338: 100–111. 10.1016/j.brainres.2010.03.103 20380821

[5] Miller BH, Wahlestedt C. MicroRNA dysregulation in psychiatric disease. Brain Res. 2010;1338: 89–99. 10.1016/j.brainres.2010.03.035 20303342 PMC2891055

[6] Xu B, Karayiorgou M, Gogos JA. MicroRNAs in psychiatric and neurodevelopmental disorders. Brain Res. 2010;1338: 78–88. 10.1016/j.brainres.2010.03.109 20388499 PMC2883644

[7] Bartel DP. MicroRNAs: target recognition and regulatory functions. Cell. 2009;136: 215–233. 10.1016/j.cell.2009.01.002 19167326 PMC3794896

[8] Iacoangeli A, Bianchi R, Tiedge H. Regulatory RNAs in brain function and disorders. Brain Res. 2010;1338: 36–47. 10.1016/j.brainres.2010.03.042 20307503 PMC3524968

[9] Amaral PP, Mattick JS. Noncoding RNA in development. Mamm Genome. 2008;19: 454–492. 10.1007/s00335-008-9136-7 18839252

[10] Krichevsky AM, King KS, Donahue CP, Khrapko K, Kosik KS. A microRNA array reveals extensive regulation of microRNAs during brain development. RNA. 2003;9: 1274–1281. 13130141 10.1261/rna.5980303 PMC1370491

[11] Chang S, Wen S, Chen D, Jin P. Small regulatory RNAs in neurodevelopmental disorders. Hum Mol Genet. 2009;18: R18–26. 10.1093/hmg/ddp072 19297398 PMC2657940

[12] Mercer TR, Dinger ME, Mariani J, Kosik KS, Mehler MF, Mattick JS. Noncoding RNAs in Long-Term Memory Formation. Neuroscientist. 2008;14: 434–445. 10.1177/1073858408319187 18997122

[13] Calin GA, Dumitru CD, Shimizu M, Bichi R, Zupo S, Noch E, et al Frequent deletions and down-regulation of micro-RNA genes miR15 and miR16 at 13q14 in chronic lymphocytic leukemia. Proc Natl Acad Sci U S A. 2002;99: 15524–15529. 12434020 10.1073/pnas.242606799 PMC137750

[14] Takamizawa J, Konishi H, Yanagisawa K, Tomida S, Osada H, Endo H, et al Reduced expression of the let-7 microRNAs in human lung cancers in association with shortened postoperative survival. Cancer Res. 2004;64: 3753–3756. 15172979 10.1158/0008-5472.CAN-04-0637

[15] Rizos E, Siafakas N, Koumarianou A, Katsantoni E, Filippopoulou A, Ntounas P, et al miR-183 as a molecular and protective biomarker for cancer in schizophrenic subjects. Oncol Rep. 2012;28: 2200–2204. 10.3892/or.2012.2052 23007659

[16] First MR. Expanding the donor pool. Semin Nephrol. 1997;17: 373–380. 9241721

[17] Kay SR, Fiszbein A, Opler LA. The positive and negative syndrome scale (PANSS) for schizophrenia. Schizophr Bull. 1987;13: 261–276. 3616518 10.1093/schbul/13.2.261

[18] Buysse DJ, Reynolds CF, Hauri PJ, Roth T, Stepanski EJ, Thorpy MJ, et al Diagnostic concordance for DSM-IV sleep disorders: a report from the APA/NIMH DSM-IV field trial. Am J Psychiatry. 1994;151: 1351–1360. 8067492 10.1176/ajp.151.9.1351

[19] Ritchie ME, Silver J, Oshlack A, Holmes M, Diyagama D, Holloway A, et al A comparison of background correction methods for two-colour microarrays. Bioinformatics. 2007;23: 2700–2707. 17720982 10.1093/bioinformatics/btm412

[20] Asikainen S, Rudgalvyte M, Heikkinen L, Louhiranta K, Lakso M, Wong G, et al Global microRNA expression profiling of Caenorhabditis elegans Parkinson's disease models. J Mol Neurosci. 2010;41: 210–218. 10.1007/s12031-009-9325-1 20091141

[21] Gehrke S, Imai Y, Sokol N, Lu B. Pathogenic LRRK2 negatively regulates microRNA-mediated translational repression. Nature. 2010;466: 637–641. 10.1038/nature09191 20671708 PMC3049892

[22] Niwa R, Zhou F, Li C, Slack FJ. The expression of the Alzheimer's amyloid precursor protein-like gene is regulated by developmental timing microRNAs and their targets in Caenorhabditis elegans. Dev Biol. 2008;315: 418–425. 10.1016/j.ydbio.2007.12.044 18262516 PMC2307910

[23] Lehmann SM, Kruger C, Park B, Derkow K, Rosenberger K, Baumgart J, et al An unconventional role for miRNA: let-7 activates Toll-like receptor 7 and causes neurodegeneration. Nat Neurosci. 2012;15: 827–835. 10.1038/nn.3113 22610069

[24] Zhao B, Han H, Chen J, Zhang Z, Li S, Fang F, et al MicroRNA let-7c inhibits migration and invasion of human non-small cell lung cancer by targeting ITGB3 and MAP4K3. Cancer Lett. 2014;342: 43–51. 10.1016/j.canlet.2013.08.030 23981581

[25] Tang H, Zhang P, Xiang Q, Yin J, Yu J, Yang X, et al Let-7 g microRNA sensitizes fluorouracil-resistant human hepatoma cells. Pharmazie. 2014;69: 287–292. 24791593

[26] Xia Y, Zhu Y, Zhou X, Chen Y. Low expression of let-7 predicts poor prognosis in patients with multiple cancers: a meta-analysis. Tumour Biol. 2014;35: 5143–5148. 10.1007/s13277-014-1663-0 24756756

[27] Patel K, Kollory A, Takashima A, Sarkar S, Faller DV, Ghosh SK. MicroRNA let-7 downregulates STAT3 phosphorylation in pancreatic cancer cells by increasing SOCS3 expression. Cancer Lett. 2014;347: 54–64. 10.1016/j.canlet.2014.01.020 24491408 PMC3972339

[28] Fu LL, Wen X, Bao JK, Liu B. MicroRNA-modulated autophagic signaling networks in cancer. Int J Biochem Cell Biol. 2012;44: 733–736. 10.1016/j.biocel.2012.02.004 22342941

[29] Jian X, Xiao-yan Z, Bin H, Yu-feng Z, Bo K, Zhi-nong W, et al MiR-204 regulate cardiomyocyte autophagy induced by hypoxia-reoxygenation through LC3-II. Int J Cardiol. 2011;148: 110–112. 10.1016/j.ijcard.2011.01.029 21316776

[30] Li ZY, Yang Y, Ming M, Liu B. Mitochondrial ROS generation for regulation of autophagic pathways in cancer. Biochem Biophys Res Commun. 2011;414: 5–8. 10.1016/j.bbrc.2011.09.046 21951851

[31] Abraham D, Jackson N, Gundara JS, Zhao J, Gill AJ, Delbridge L, et al MicroRNA profiling of sporadic and hereditary medullary thyroid cancer identifies predictors of nodal metastasis, prognosis, and potential therapeutic targets. Clin Cancer Res. 2011;17: 4772–4781. 10.1158/1078-0432.CCR-11-0242 21622722

[32] Sarver AL, Li L, Subramanian S. MicroRNA miR-183 functions as an oncogene by targeting the transcription factor EGR1 and promoting tumor cell migration. Cancer Res. 2010;70: 9570–9580. 10.1158/0008-5472.CAN-10-2074 21118966

[33] Babashah S, Soleimani M. The oncogenic and tumour suppressive roles of microRNAs in cancer and apoptosis. Eur J Cancer. 2011;47: 1127–1137. 10.1016/j.ejca.2011.02.008 21402473

[34] Tan L, Yu JT, Tan MS, Liu QY, Wang HF, Zhang W, et al Genome-wide serum microRNA expression profiling identifies serum biomarkers for Alzheimer's disease. J Alzheimers Dis. 2014;40: 1017–1027. 10.3233/JAD-132144 24577456

[35] Di Cello F, Hillion J, Hristov A, Wood LJ, Mukherjee M, Schuldenfrei A, et al HMGA2 participates in transformation in human lung cancer. Mol Cancer Res. 2008;6: 743–750. 10.1158/1541-7786.MCR-07-0095 18505920 PMC3086547

[36] Day E, Poulogiannis G, McCaughan F, Mulholland S, Arends MJ, Ibrahim AEK, et al is a candidate driver oncogene on 13q34 in colorectal cancer. Int J Exp Pathol. 2013;94: 203–211. 10.1111/iep.12021 23594372 PMC3664965

[37] Le HT, Sorrell AM, Siddle K. Two isoforms of the mRNA binding protein IGF2BP2 are generated by alternative translational initiation. PLoS One 2012;7: e33140 10.1371/journal.pone.0033140 22427968 PMC3299737

[38] Thumar J, Shahbazian D, Aziz SA, Jilaveanu LB, Kluger HM. MEK targeting in N-RAS mutated metastatic melanoma. Mol Cancer. 2014;13: 45 10.1186/1476-4598-13-45 24588908 PMC3945937

[39] Kean MJ, Williams KC, Skalski M, Myers D, Burtnik A, Foster D, et al VAMP3, syntaxin-13 and SNAP23 are involved in secretion of matrix metalloproteinases, degradation of the extracellular matrix and cell invasion. J Cell Sci. 2009;122: 4089–4098. 10.1242/jcs.052761 19910495

[40] Aherne A, Kennan A, Kenna PF, McNally N, Lloyd DG, Alberts IL, et al On the molecular pathology of neurodegeneration in IMPDH1-based retinitis pigmentosa. Hum Mol Genet. 2004;13: 641–650. 14981049 10.1093/hmg/ddh061

