# Correction: Let-7, Mir-98 and Mir-181 as Biomarkers for Cancer and Schizophrenia

**DOI:** 10.1371/journal.pone.0135863

**Published:** 2015-08-12

**Authors:** Emmanouil Rizos, Nikolaos Siafakas, Eleni Katsantoni, Eleni Skourti, Vassilios Salpeas, Ioannis Rizos, James N. Tsoporis, Anastasia Kastania, Anastasia Filippopoulou, Nikolaos Xiros, Demetrios Margaritis, Thomas G. Parker, Charalabos Papageorgiou, Vassilios Zoumpourlis

There is an error in the title of this article: “Mir-181” should be “Mir-183.” The correct title is: Let-7, Mir-98 and Mir-183 as Biomarkers for Cancer and Schizophrenia. The correct citation is: Rizos E, Siafakas N, Katsantoni E, Skourti E, Salpeas V, Rizos I, et al. (2015) Let-7, Mir-98 and Mir-183 as Biomarkers for Cancer and Schizophrenia. PLoS ONE 10(4): e0123522. doi:10.1371/journal.pone.0123522

## References

[1] Rizos E, Siafakas N, Katsantoni E, Skourti E, Salpeas V, Rizos I, et al (2015) Let-7, Mir-98 and Mir-181 as Biomarkers for Cancer and Schizophrenia. PLoS ONE 10(4): e0123522 doi: 10.1371/journal.pone.0123522 25856466 10.1371/journal.pone.0123522 PMC4391828

